# Numerical Analysis of the *ACL*, with Sprains of Different Degrees after Trauma

**DOI:** 10.1155/2021/2109348

**Published:** 2021-07-24

**Authors:** Rodrigo Arturo Marquet-Rivera, Guillermo Urriolagoitia-Sosa, Beatriz Romero-Ángeles, Rosa Alicia Hernández-Vázquez, Octavio Alejandro Mastache-Miranda, Salvador Cruz-López, Arturo Torres-Yáñez, Guillermo Urriolagoitia-Calderón

**Affiliations:** ^1^Instituto Politécnico Nacional, Escuela Superior de Ingeniería Mecánica y Eléctrica, Sección de Estudios de Posgrado e Investigación, Unidad Profesional Adolfo López Mateos “Zacatenco”, Avenida Instituto Politécnico Nacional, s/n Edificio 5, 2do. Piso, Col. Lindavista, C.P. 07738 Ciudad de México, Mexico; ^2^Universidad Politécnica del Valle de México, Departamento de Mecatrónica, Av. Mexiquense s/n Esquina Av. Universidad Politécnica, Col. Villa Esmeralda, Tultitlán, C.P. 54910 Estado de México, Mexico; ^3^Instituto Politécnico Nacional, Unidad Profesional Interdisciplinaria en Energía y Movilidad, Unidad Profesional Adolfo López Mateos “Zacatenco”, Av. Luis Enrique Erro S/N, Col. Lindavista, C.P. 07338 Ciudad de México, Mexico

## Abstract

Nowadays, cruciate ligament injuries have increased in incidence, since practicing a sport or physical activity has become a trend in current societies. Although this lifestyle generates multiple benefits, as a consequence, injury has also increased. Due to its nature and complexity, the ligaments of the knee are those that are most frequently affected, mainly the *ACL* (anterior cruciate ligament). This tissue reacts to overexertion or movements out of range, either caused by the exercise itself or caused by trauma caused by the practice of physical activity, causing various degrees of sprain. Whatever the etiology of these injuries, they will require a therapy indicated for each degree of injury. This therapy initially entails immobilization of the affected area and later; physical therapy will be required to a lesser or greater degree. Commonly, in the physiotherapy of these injuries, rehabilitation exercises are prescribed, where the physiotherapist asks a patient to use equipment with an estimated weight. However, the effectiveness of a generalized therapy in this way does not always give the expected results. This is related to the fact that these therapies are standardized and do not consider some factors such as the remaining muscle fibres that are not directly affected by the sprain, which does not mean that they should not be considered. Therefore, in the present work, a biomodel of a human knee has been developed and used to evaluate numerically how the *ACL* acts under an external load, when there are different degrees of injuries, caused by trauma. Four case studies were considered: Case 1 (control case) where the *ACL* is healthy, Case 2 where the *ACL* presents a 1^st^-degree sprain, Case 3 where the *ACL* presents a 2^nd^-degree sprain, and finally Case 4 where the *ACL* presents a 3^rd^-sprain grade. After performing the analyses, in the control case, it was found that it presents a balance between tensile and compressive stresses. While in the 4th case, the most critical tensile stress decreases while compression stresses increase. This shows that the ligament, having considerable damage, no longer works as it should and can eventually damage the collateral structures. It was found that, when there was a sprain, where the continuity of the ligament is compromised, a second torsional moment occurs in the ACL which causes the tissue fibres not to act according to their normal physiology or in a healthy state. The results obtained from the present study provide the possibility of predicting where the following injuries will occur by considering the *von Mises* failure criterion. Likewise, they will allow to improve the therapeutic procedures considering not only the injured structure but also the system as a whole.

## 1. Introduction

Nowadays, the culture of sports is more than a fashion; it has become a lifestyle. Sedentarism causes diverse affections in people's health; this is why, practicing a sport, rather than a hobby, is a discipline that is part of everyday life [1, 2]. Although the benefits are multiple, there are as well negative results involved, and physical injuries have also increased, as a result of this active life [3, 4].

Studies estimate that 80% of physical injuries that occur during training and the practice of a sport occur in soft tissues (muscles, tendons, ligaments, and joints). The remaining 20% correspond to fractures (hard tissue) or damage to internal organs [5]. Of these injuries, it has been found that the anatomical structures with the highest incidence are the knee (45.5%), ankle (9.8%), and shoulder (7.7%). From these percentages, 53.9% involve soft tissues [6]. Concerning soft tissues, the lesions can range from bruises, abrasions, and cramps to muscle strains and sprains that in 75% of cases do not require medical treatment. Between 30 and 50% of these injuries are produced by excessive use of these soft tissues [7]. This reacts to injuries: incapacity for competitors of professional athletes as well for common people who exercise or practice sports recreationally [8]. It should be noted that sprains in the joints are the most common injuries and those of the knee in particular represent 25 to 40% of medical consultations derived from sports injuries [9].

Sprains are classified into 3 different degrees of damage. In the first degree, some microfractures occur along the ligament, in the second degree, it is possible to observe a significant fracture part of the element, and in the third degree, there is a total separation or rupture of the ligament [10]. When any of these injuries occurs, the general therapy is through immobilization of the joint and physical rehabilitation or physiotherapy [11–13] regardless of whether or not they require surgical intervention, as it is applied in any case. Physical therapy uses physical agents to treat injuries: temperature variation, radiofrequency, and laser, among others but, more commonly, specific movements with or without weight [14–18]. The latter base their principles on mechanotransduction, mechanical stimulation through these exercises, so that the body is able to regenerate the injury by itself [19–22]. However, each person has different capacities; they do not have the same motor or physical capabilities, so using a standardized load for all patients may not be quite appropriate. This has meant that in practice, therapists may not consider that the mechanical stresses generated by the same loads or forces exerted during rehabilitation could eventually lead to damage the ligament [23]. Such stresses may fall into a range in which, rather than being beneficial, they prove to be harmful. This could be due to the mechanical limit of living tissues (muscles, bones, ligaments, etc.) being exceeded, as a consequence of a not bad intentioned, but inadequate indication by the therapist or an inadequate follow-up of the patient, exceeding the indicated weight or the speed of movement. This may result, as in the case of a lesion in the *ACL*, that patients are unable to fully perform the natural movements of the knee such as flexion-extension, adduction-abduction, and rotations [24]. This ligament is more frequently injured in athletes, due to sudden deceleration and/or abrupt turns when running, jumping, etc. To diagnose this pathology, medical staff help themselves with different tests already established in medicine such as Pivot Shift, Lachman, previous drawer,1 and technological advances in the area of Imaging (Computed Tomography, Magnetic Resonance, etc.) [25, 26].

Several biomedical studies of the *ACL* have been carried out, which have allowed the understanding of the functioning and behavior of this tissue [27–31]. To perform these studies, it should implement the application of numerical analysis using the Finite Element Method (*FEM*) [32] and apply the various lesions that occur in this ligament during training, sports, etc., such as FEM analysis, based on experiments with cadaver bones [33], of the effect of bone tunnel sites on the kinetics and distribution of stress and functional results [34], and investigation of the biomechanics and kinematics of the knee to optimize graft placement, among others [35]. With this type of analysis, it is possible to observe the stress distribution generated by a simulated load, which emulates a physical trauma, and, in this way, know the effects of the external agent can induce damage to the injured tissue. This tool can be very useful to safely determine an allowable capacity of the tissue.

The present work is a proposal on how to use a numerical analysis and simulation of the tensions generated during a physical trauma with a new perspective. Consider not only damaged tissue but also the remaining healthy tissue, with a high biofidelity biomodel. In addition, in this way, it is possible to implement improvements in traditional therapeutics.

## 2. Materials and Methods

In the present work, four cases of study were carried out by applying a numerical simulation method to a human knee using biomodels generated with a methodology developed by the authors in previous works [36–38]. The detailed development of these biomodels and their validation are widely described in [38]. In Case 1, the biological elements do not present any kind of physical injury, so this corresponds to the control case. In Case 2, the *ACL* presents microfractures, corresponding to a 1^st^-degree lesion or sprain of 1^st^ degree. Case 3 presents a 2^nd^-degree injury with a partial but considerable ligament rupture or 2^nd^-degree sprain. Finally, Case 4 presents an almost total rupture (3^rd^-degree injury or 3^rd^-degree sprain).

The biomodel was developed from Magnetic Resonance (*MR*), in which *DICOM* image files were imported to a *CAM/CAD* computer program that interprets these images to obtain the *3D* model. Once the *DICOM* file import has already been done, it is necessary to define the area of interest for the work. With the program's tool, create a mask, then select the range values of the threshold that corresponds to each tissue, and finally refine the generated area to eliminate the excessive areas. This program stores the biomodel file to a ^∗^.*stl* file format. This file is exported to a *CAD/CAM/FEM* program.

**Figure 1:**
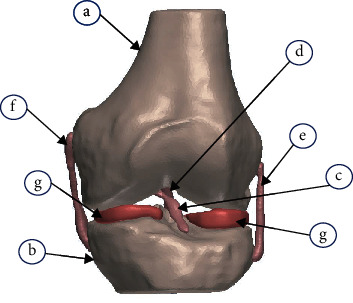
Biomodel of high biofidelity of the knee. (a) Cortical bone of the last third of the femur. (b) Cortical bone of the tibial plateau. (c) Anterior cruciate ligament (*ACL*). (d) Posterior cruciate ligament (*PCL*). (e) External lateral ligament (*ELL*). (f) Internal lateral ligament (*ILL*). (g) Meniscus.

Figure 1 shows a biomodel of a human knee which is subjected to a compression load of 500 N, exerted on the tibia simulating an impact [36–40]. With this simulation, we can obtain the stresses that are exerted in this patellar joint after an agent of that nature. This analysis will be performed for the joint when the *ACL* presents sprain-type lesions in the different degrees of injury.

On the numerical analysis, the contacts between the tissues must be contemplated.  Table 1 lists the 14 contacts included in the interaction of all the biological elements of this biomodel.

All the contacts are *bonded* type, except for contacts 6 and 7, which are between the cortical bone of the femur and the meniscus, which are *frictionless contact* [34–41]. Because of the joint physiology of these tissues, there is a movement. Contacts were established in this manner, as doing so is an accepted concession. In most of this kind of jobs, it is required to establish the contacts between these tissues, in this manner. This can be found in basic literature.

**Figure 2:**
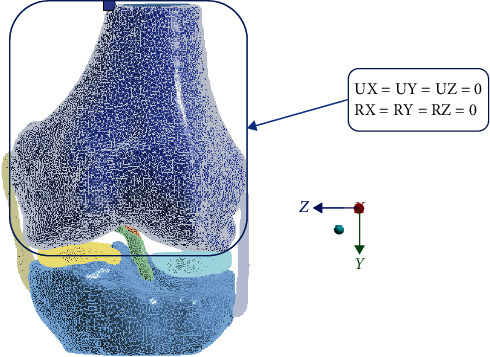
Restriction of movement in the cortical bone of the last third of the femur.

**Figure 3:**
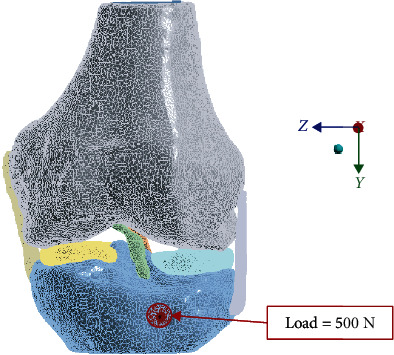
Loading action (compression).

For the numerical simulation, the Finite Element Method was used. The biomodels' tissues are considered materials that present a linear, elastic, continuous behavior and whose internal structure is isotropic and homogeneous. To be able to perform this simulation, it is necessary to establish boundary conditions and restrict movement. Therefore, we proceed to restrict the displacements and rotations in the directions of the *X*-, *Y*-, and *Z*-axes in the femur as shown in Figure 2, and the force exerted is placed on the tibia as shown in Figure 3. The properties of the materials considered for the simulation are presented in Table 2.

Table 3 shows the characteristics of the biomodels generated for the numerical analysis.

Finally, the normal stresses are obtained in the different axes (*X*, *Y*, *Z*), with which the magnitude and direction (tension or compression) generated by the external agent on the ACL can be determined, in addition to the von Mises stresses. This criterion is applied as a conservative theory of failure (which is mainly applicable to ductile materials) and as a single nondirectional value that allows to have a global criterion on the load at each point of the ACL, since it is obtained through von Mises, the strain energy.

## 3. Results

### 3.1. Case 1 (Control)

**Figure 4:**
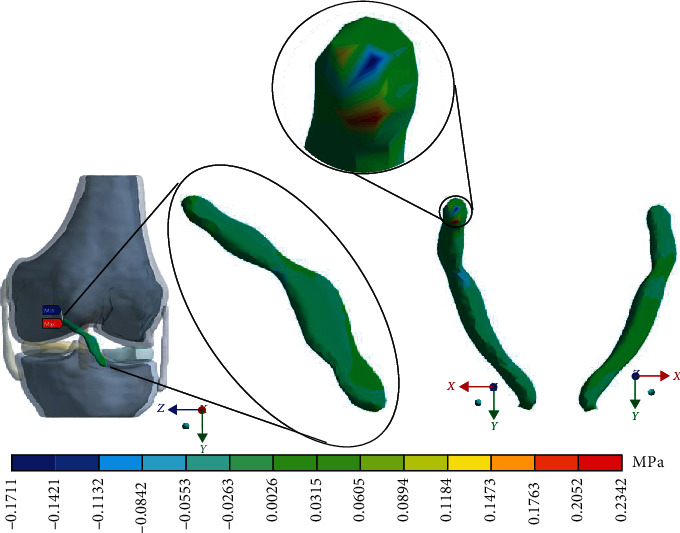
Normal stress presented in the *X*-axis in the control case.

For the control case with the healthy *ACL*, Figure 4 shows the normal stresses on the *X*-axis.

#### 3.1.1. Results on the *Y*-Axis

**Figure 5:**
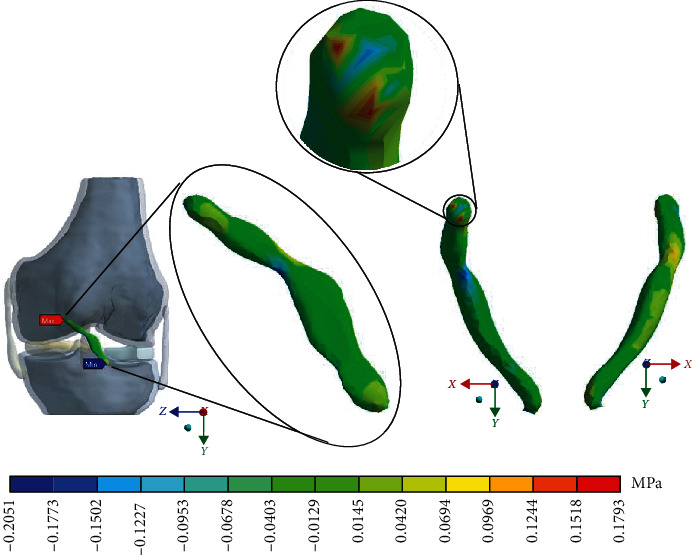
Normal stress presented in the *Y*-axis in the control case.

For the control case with the healthy *ACL*, Figure 5 shows the normal stresses on the *Y*-axis.

#### 3.1.2. Results on the *Z*-Axis

**Figure 6:**
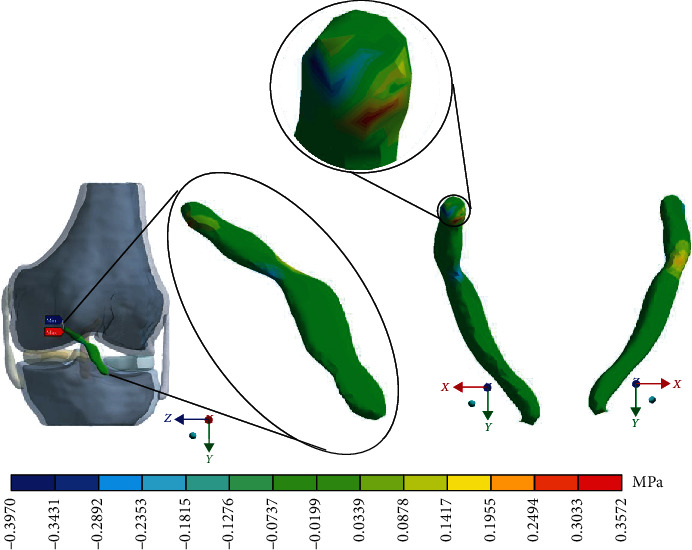
Normal stress presented in the *Z*-axis in the control case.

For the control case with the healthy *ACL*, Figure 6 shows the normal stresses on the *Z*-axis.

#### 3.1.3. Results in *von Mises*

**Figure 7:**
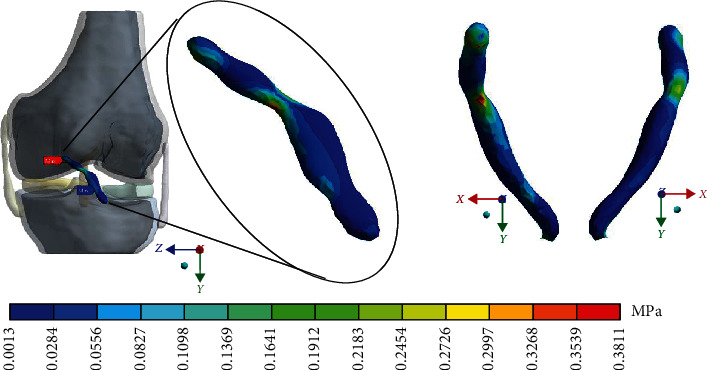
*von Mises* stress presented in the control case.

For the control case with the healthy *ACL*, Figure 7 shows the stress of *von Mises.*

### 3.2. Case 2 (*ACL* Sprain of 1^st^ Degree)

**Figure 8:**
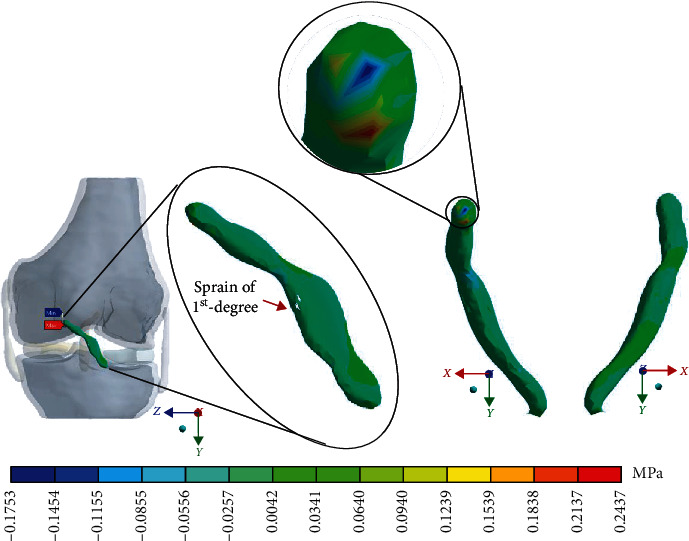
Normal stress presented in the *X*-axis in Case 2.

For Case 2 where the *ACL* presents a 1^st^-degree sprain or microruptures, Figure 8 shows the normal stresses on the *X*-axis.

#### 3.2.1. Results on the *Y*-Axis

**Figure 9:**
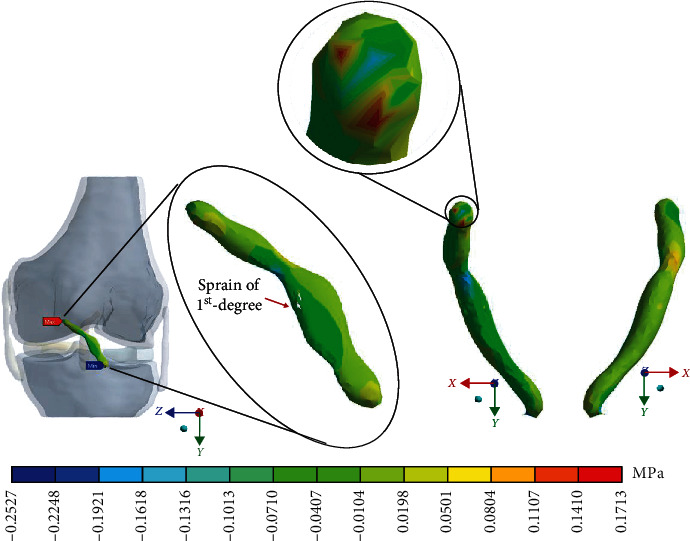
Normal stress presented in the *Y*-axis in Case 2.

For Case 2 where the *ACL* presents a 1^st^-degree sprain or microruptures, Figure 9 shows the normal stresses on the *Y*-axis.

#### 3.2.2. Results on the *Z*-Axis

**Figure 10:**
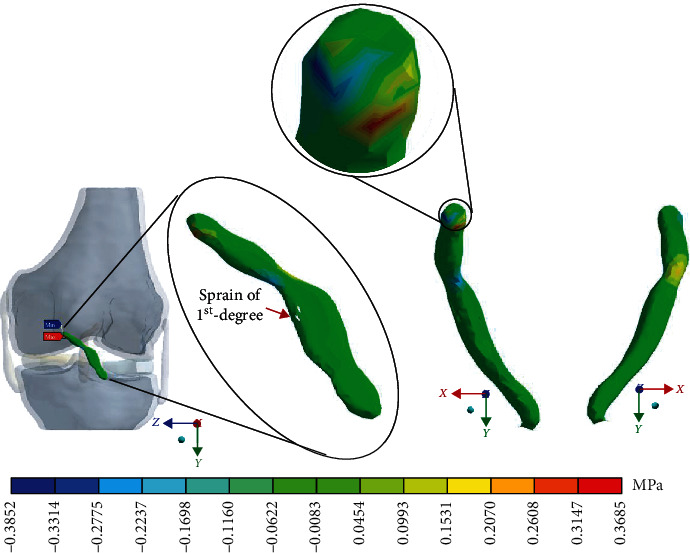
Normal stress presented in the *Z*-axis in Case 2.

For Case 2, where the *ACL* presents a 1^st^-degree sprain or microruptures, Figure 10 shows the normal stresses on the *Z*-axis.

#### 3.2.3. Results in von Mises

**Figure 11:**
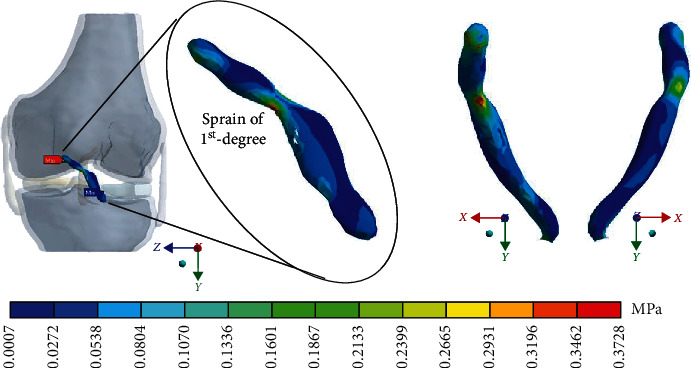
*von Mises* stress presented in Case 2.

For Case 2 where the *ACL* presents a 1^st^-degree sprain or microruptures, Figure 11 shows *von Mises* stress.

### 3.3. Case 3 (*ACL* Sprain of 2^nd^ Degree)

**Figure 12:**
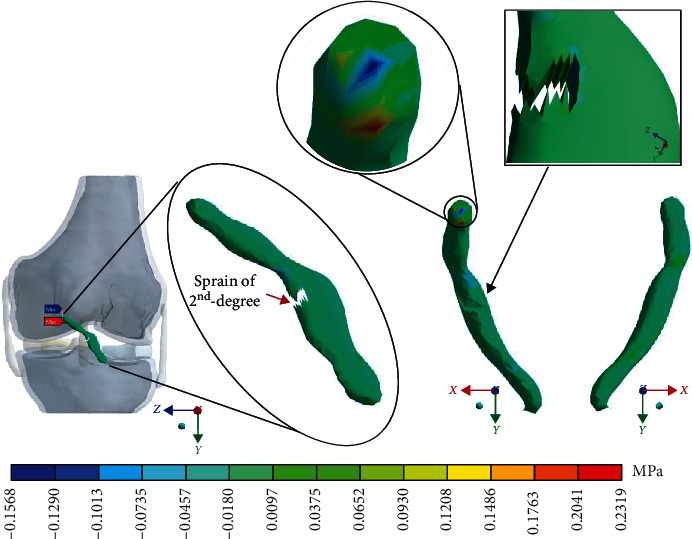
Normal stress presented in the *X*-axis in Case 3.

For Case 3 where the *ACL* presents a 2^nd^-degree sprain or a partial rupture, Figure 12 shows the normal stresses on the *X*-axis.

#### 3.3.1. Results on the *Y*-Axis

**Figure 13:**
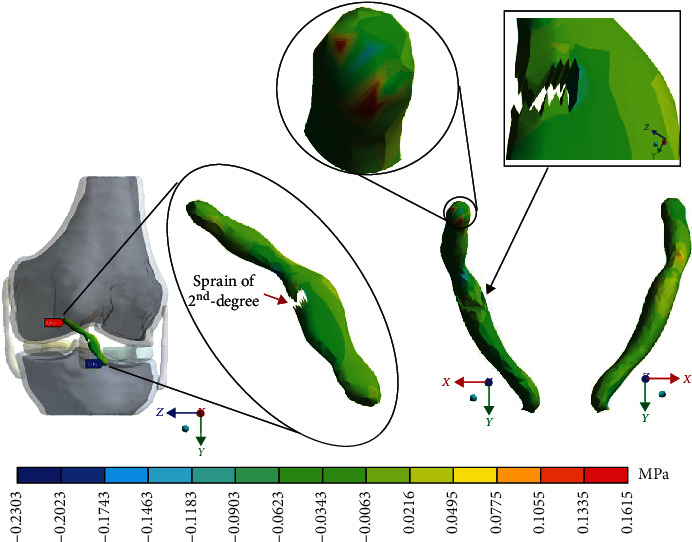
Normal stress presented in the *Y*-axis in Case 3.

For Case 3 where the *ACL* presents a 2^nd^-degree sprain or a partial rupture, Figure 13 shows the normal stresses on the *Y*-axis.

#### 3.3.2. Results on the *Z*-Axis

**Figure 14:**
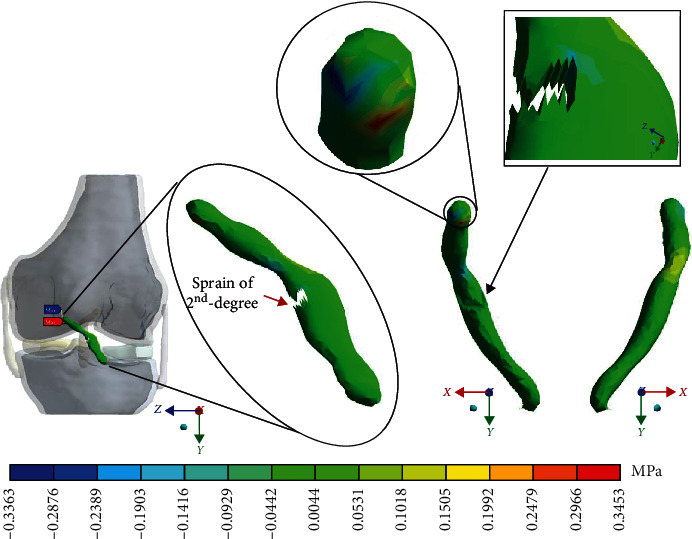
Normal stress presented in the *Z*-axis in Case 3.

For Case 3 where the *ACL* presents a 2^nd^-degree sprain or partial rupture, Figure 14 shows the normal stresses on the *Z*-axis.

#### 3.3.3. Results in von Mises

**Figure 15:**
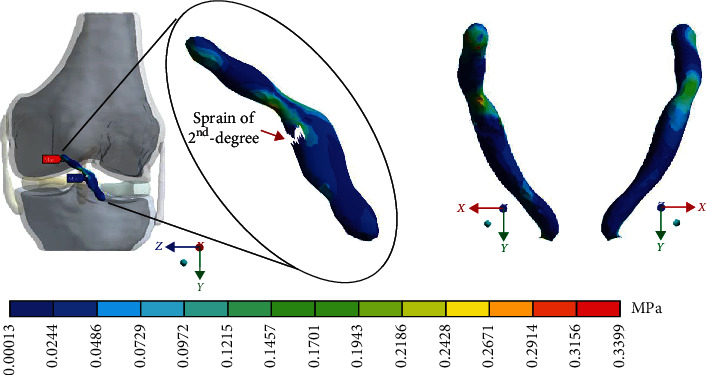
*von Mises* stress presented in Case 3.

For Case 3 where the *ACL* presents a 2^nd^-degree sprain or a partial rupture, Figure 15 shows the *von Mises* stresses.

### 3.4. Case 4 (*ALC* Sprain of 3^rd^ Degree)

**Figure 16:**
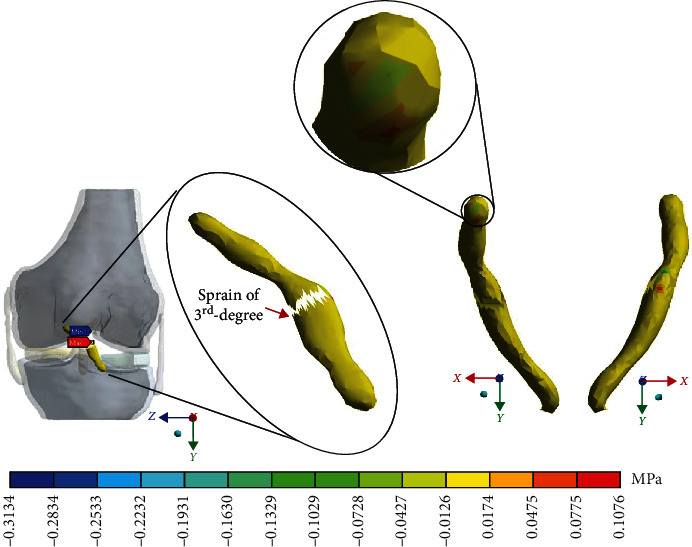
Normal stress presented in the *X*-axis in Case 4.

For Case 4 where the *ACL* presents a 3^rd^-degree sprain or an almost total or total rupture, Figure 16 shows the normal stresses on the *X*-axis.

#### 3.4.1. Results on the *Y*-Axis

**Figure 17:**
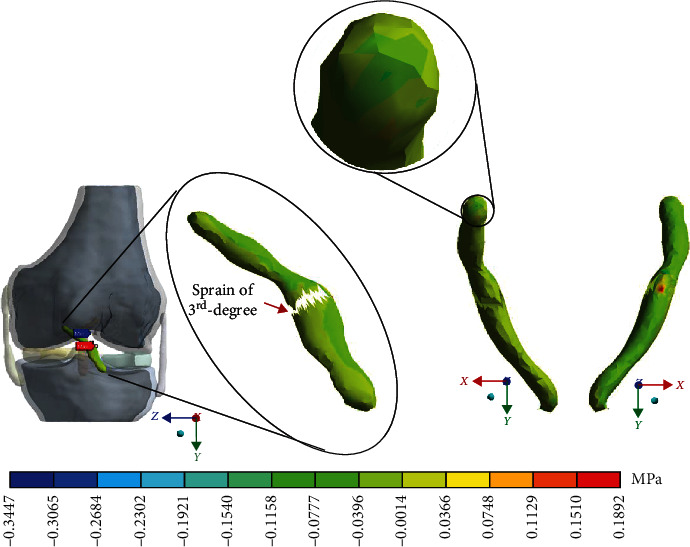
Normal stress presented in the *Y*-axis in Case 4.

For Case 4 where the *ACL* presents a 3^rd^-degree sprain or an almost total or total rupture, Figure 17 shows the normal stresses on the *Y*-axis.

#### 3.4.2. Results on the *Z*-Axis

**Figure 18:**
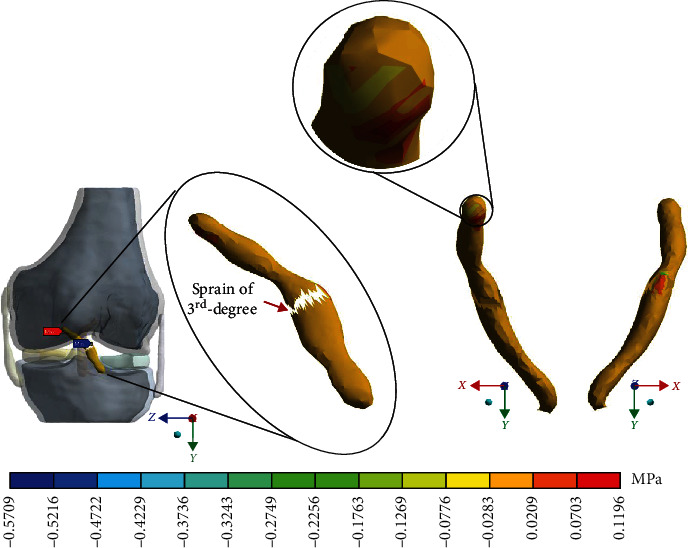
Normal stress presented in the *Z*-axis in Case 4.

For Case 4 where the *ACL* presents a 3^rd^-degree sprain or an almost total or total rupture, Figure 18 shows the normal stresses on the *Z*-axis.

#### 3.4.3. Results in von Mises

**Figure 19:**
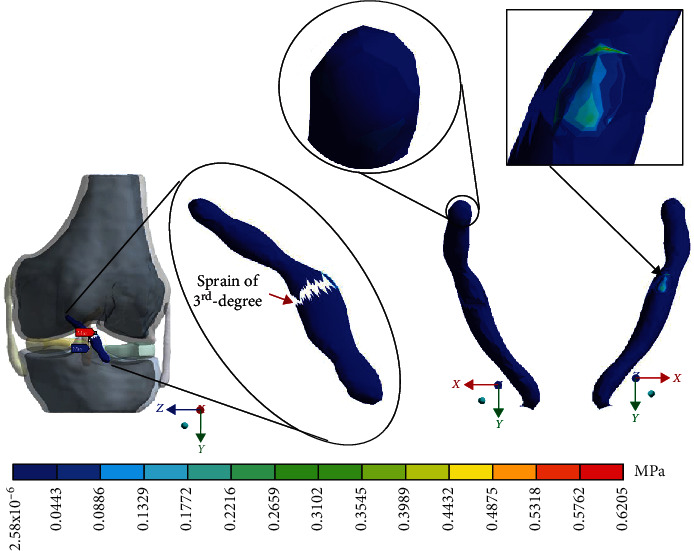
*von Mises* stress presented in Case 4.

For Case 4, where the *ACL* presents a 3^rd^-degree sprain or an almost total or total rupture, Figure 19 shows the *von Mises* stresses.

Table 4 shows a comparison of the maximum and minimum values in compression and tensile stresses that occur in the *ACL*. Consider a healthy *ACL* as reference (Case 1), until to the most drastic case, the third-degree sprain, with an almost total rupture (Case 4).  Table 5 shows a comparison of the *von Mises* stresses for each case.

## 4. Discussion

The knee is one of the most complex and specialized joints in the human body. In its physiology, many movements and/or rotations are involved that perform in a harmonious way in the different structures such as hard and soft tissues. This causes the specific function of each anatomical element, which in some cases is antagonistic to each other, but harmonious at the same time, to generate a varied series of positive/negative stresses and deformations. When an imbalance occurs in this harmonic and synergistic functioning, as in the case of injuries and pathologies, some considerations go beyond simply applying a therapy to the damaged structure.

The methodology used in this work is based on previous methodologies whose approach to reality is quite well accepted. The proposed methodology uses high biofidelity biomodels that allow a greater approximation to reality. The comparison of the values obtained from the previous studies is already established in the basic literature where they were compared with mechanical tests. It is important to remember that the performance of mechanical tests on biological tissues cannot be the same as reality due to conditions such as the loss of water in the tissues (dehydration, etc.) However, it has been possible to establish the same parameters that are congruent with the results obtained in this study.

The Contact Manager® subprogram was used, which is of simple and common use in this type of analysis, typical of the ANSYS® software. Most software that works with finite elements has a subprogram of this type. Through these subprograms, the contacts that exist between the elements of the model are established. With this subprogram, Contact Manager®, it is possible to establish the contact element (contact body) and the element that receives such contact (target body), which is represented by the basic colors red (Contact body) and blue (Target body). In Table 1, in addition to clearly showing the contacts with these colors, mention is made of the elements involved.

As already mentioned in the methodology, the analysis was carried out considering that the materials have a linear, homogeneous, continuous, and isotropic behaviour. The discretized mesh is semicontrolled with high-order tetrahedral elements. All these attributes are commonly used when performing biomodelling and numerical analysis on biological tissues and organs [22, 37, 43–50].

With this kind of methodologies and attributes which were used to carry out this work, there is a great variety of manuscripts in the literature, where FEM analyses have been carried out, which are valid and feasible to use in any organ or tissue of the human body. Soft tissue work can be found such as in the ligaments [51], meniscus [52], veins [53], brain, and heart [54], among others [55, 56]; in hard tissues such as the skull bones [57], vertebrae [58], and even smaller anatomical structures, but with great geometric complexity such as teeth [37]; and in other joints such as the temporomandibular joint and mandible [39] and shoulder [59], among others. However, there are few studies with a conjunction of so many tissues involved in the same FEM analysis as the one in the present study.

With the results obtained in the present work, it is apparent that, regardless of the fact that the degree of injury the ligament does not present a total rupture, the residual fibres (tissue that was not injured or torn) show results that should be considered important for both the mechanical behaviour and decision of the therapy to be applied. This is especially evident in the case of the third-degree sprain.

In the numerical analysis corresponding to the third-degree of injury, it is possible to observe that, at the area of the residual tissue, being less than the area of contact, between tissues (where the ligament joins the bone), the maximum and minimum stresses are presented at the injured region.

**Figure 20:**
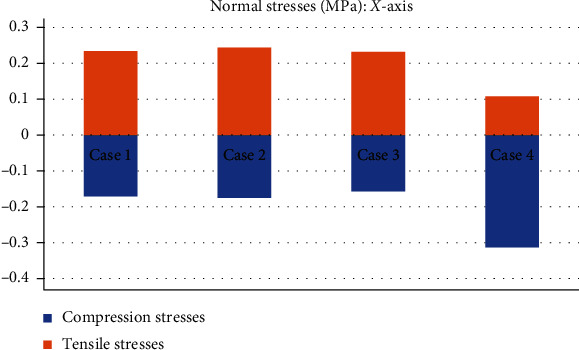
Comparison between compression stresses and tensile stresses (MPa) on the *X*-axis.

This seems be logical, as the residual tissue, being undamaged, can continue to fulfil its normal physiological function. In the same way, it would be expected that the surrounding superior and inferior extremities to the lesion, when continued to attach to the bone, continue to function, as shown in Figures 12–15 corresponding to the 2^nd^ sprain. However, in the 3^rd^-degree sprain, those surrounding areas are not functioning as shown in Figures 16–19. This causes the residual uninjured tissue, therefore healthy, to function outside of its range, which requires it to support a greater work than should the physiologically fulfil. However, for the 3^rd^-degree sprain, those surrounding areas are not functioning. This causes the uninjured residual tissue, therefore healthy, to function outside its range and/or required to support a bigger load for which it is physiologically expected to endure. This is of the utmost importance since it is proposed to consider that the therapy is not only for the area that was injured but also for the area that should be considered, for healthy tissue, that is functioning outside of its range and in accordance with its nature, since it is also supporting compressions and not tensions that is the way it should work. Besides, the fibres that make up the residual zone, being subjected to these out-of-range agents, could already be in a state of elastoplasticity, also considering that, although the rupture zone is in relative repose for regeneration, where new fibres will proliferate, the healthy residual tissue will not be at rest, so it will continue to work, which will cause it to become a material with a previous history not totally healthy. Table 4 and Figure 20 show a comparison between the values obtained from compression stresses and tensile stresses on the *X*-axis of each case study. In Case 1, where the ligament is healthy, both stresses, compression and tensile, have similar values. In Case 2, stresses in tensile are greater than stresses in compression. For Case 3, compression stresses are smaller than tensile stresses. Finally, in Case 4, compression stresses are even greater than tensile stresses. In this case, the ligament function is not normal.

**Figure 21:**
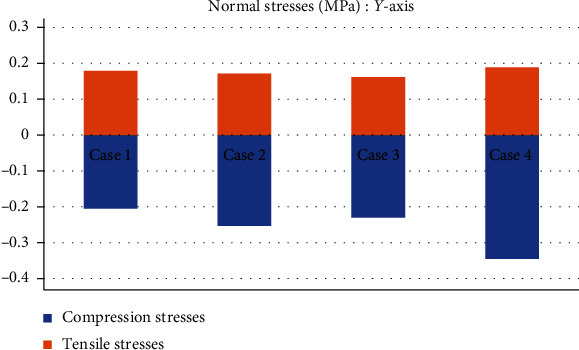
Comparison between compression stresses and tensile stresses (MPa) on the *Y*-axis.

Table 4 and Figure 21 show a comparison between those values obtained for compression and tensile stresses on the *X*-axis of each study case. In Case 1, a similar phenomenon occurs between the stresses as they occur on the *X*-axis, and both stresses, compression and tensile, have equivalent values. In Case 2, in the same way, compression stresses are greater than tensile stresses. For Case 3, by contrast as presented on the *X*- and *Y*-axis, compressive stresses are greater than tensile stresses. Finally, in Case 4 and by contrast for the *X*- and the *Y*-axis, compressive stresses are smaller than tensile stresses.

**Figure 22:**
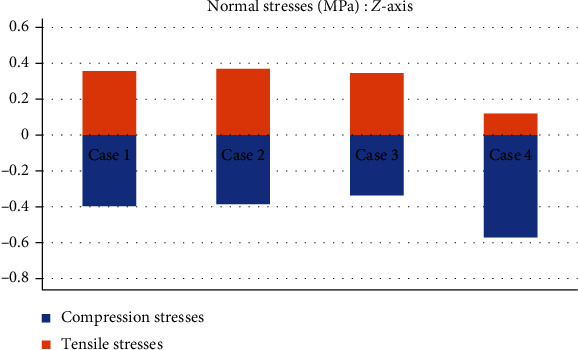
Comparison between compression stresses and tensile stresses (MPa) on the *Z*-axis.

Table 4 and Figure 22 show a comparison between the values obtained for compression and tensile stresses on the *Z*-axis, for each case study. Again, in Case 1, there is a similar phenomenon between stresses as they occur in the *X*- and *Y*-axes in which both tensile and compression stresses have equivalent values. For Case 2, compression stresses are even greater than tensile stresses. In Case 3, something similar happens wherein both tensile and compression stresses have equivalent values. Finally, in Case 4, compression stresses are much greater than tensile stresses.

**Figure 23:**
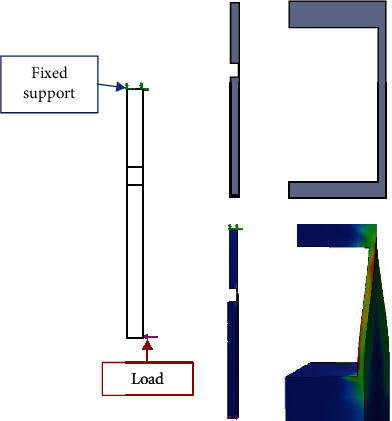
Example of torsion and flexion that occur in structures where there is no uniform structural continuity.

Based on this, it is possible to determine that the natural movement of the knee is a flexion-extension rotation, which goes from front to back (first moment). When the ligament is broken and its structural integrity is lost, leaving a few healthy fibres, the effects of the force exerted on the tibia changes and a second moment are generated on the *ACL*, which causes shear stresses. This second moment is caused by the rupture of the ligament itself because in the area where healthy fibres remain, the movement of the tibia, after being affected by the external agent, pushes the ligament, in such a way that, in the area where the rupture of the ligament fibres occurs, having to lose their continuity and, therefore, their insertion site (bone ligament contact), the residual fibres twist (flexion-torsion: combined stresses). To exemplify the phenomenon that is occurring in the *ACL*, in Figure 23, it is possible to observe a column that lost its continuity. This column is fixed at the top, and a load is applied to the base. The column behaves as the ligament of this study does. It suffers a flexion and a twist.

This torsion behaviour had not been reported in a previous work. This could be because only the rupture of the ligament was considered a phenomenon to be studied, without considering the repercussions of the structural loss of the ligament fibres and the residual fibres that did not suffer rupture.

It is difficult to be able to make a comparison of results with other studies, although there are some similar ones, such as Vairis A., Stefanoudakis G., Petousis M., Vidakis N., Tsainis AM, Kandyla B., where they investigated the mechanical behaviour of the knee joint by calculating stresses and displacements of an intact ACL, a poor ACL, and a rebuilt ACL FE model [60]. The conditions to carry them out differ from the present work. Mainly, in our work, the biomodels are of high biofidelity, and the remaining healthy tissue is considered, a factor not considered in previous studies.

With the numerical analysis such as the one presented in this work, although it is not possible to establish exactly the magnitude of the damage or the number of fibres that exceeded the elasticity range, it is possible to predict the location of the possible areas of healthy residual tissue in that there is even a history of previous injury; in this way, it is possible to deduce where future injuries may occur, as it occurs with a material with a previous history.

This is of great help to design therapeutics not only for injured and subsequently regenerated tissue but also for treatments that allow correcting to a certain extent the demands to which healthy residual tissue was subjected and thus prevent therapeutic failure in the future. In most cases, the relapse will occur in the tissue that was healthy and that was subjected to the demands of compensating the function of the injured tissue.

This type of analysis makes it possible to observe how the tissue behaves at macro- and microlevels: how the entire tissues or by zones work and react (tension and compression), their fibres and even the behaviour of their insertions or aponeurosis. With this, it has also been possible to determine if a tissue is performing a movement or work that does not correspond to its nature. By knowing the behaviour that is taking place within the tissue, the treatments for its rehabilitation can be better defined. Although there are many treatments, these are general, and on some occasions, they may be leaving remnants in the form of residual stress in some fibres of the tissues that, in situations where the tissues are subjected to out-of-range movements or overstress, they may fail. On the other hand, the affected area may be rehabilitating and leaving remnants in the adjacent anatomical structures. This type of analysis can be predictive, so instead of having new lesions, existing treatments can be complemented, so better results can be obtained. With the interdisciplinarization of health personnel with engineers, this type of injury can be approached with a greater knowledge of how tissues behave as materials, the movement they perform, and the stress remnants in healthy fibres.

## 5. Conclusions

The various physical therapies for treating ACL injuries have evolved and provided better results. However, when it comes to injuries such as the second- or third-degree sprains, therapy may not be as effective.

The rate of patients who report feeling that knee function is not the same after injury or that discomfort persists, even with daily activities, is high [26]. Thanks to the results obtained in the present work, it is possible to affirm that this is due to the fact that the therapy only focuses on immobilization that allows the regeneration of the injured or lost tissue, without considering the adjacent or remnant where the injury occurred. When exercises with specific loads and devices are indicated, it is done in a generalized way, hoping that both the new tissue and the regenerated tissue of the injury will have the possibility of recovering its function, again without considering the remaining tissues, due to the injury itself injury and seeking to compensate for the lost function of the injured tissues, were overstretched.

As already mentioned, these classic therapies that only consider damaged tissue can be successful. However, it is probable that the incomplete recovery of the patient is since residual tissue is not considered within the treatment, which was forced to work outside its range to compensate for the lack of tissue that was regenerating. Therefore, the following conclusions can be concluded:

1. The interdisciplinary work of physiotherapy, sports medicine, and biomechanics is necessary, so that the appropriate therapeutics can be designed for each case through interdisciplinary teams
2. From a medical point of view, physiotherapies seek to heal damaged tissue, which is the right thing to do. However, healthy tissue must also be treated, due to the changes that occur to compensate for the lack of tissue function that is not fulfilling its physiology
3. The surrounding healthy tissues suffer changes in their mechanical properties when trying to compensate for the presence of the lesion. Therefore, the entire system to which they belong (in this case, the knee and gait) will be affected in their normal function
4. It is known that one of the functions of the *ACL* is to avoid excessive axial rotation in the tibia over the femur. With the present work, it is demonstrated that, with the 2nd- and 3rd-degree sprains, this axial rotation cannot be limited as it should be
5. Further studies are necessary on the stresses and deformations suffered by the ligament fibres in the presence of the different injuries, as well as their cellular response (mechanobiology).
6. The numerical analysis by means of the FEM allows the prediction of areas of risk of residual tissue, to be considered within the therapy and not with this, reducing the failed treatments and the sequelae

With all this, the possibility of designing better treatments and therapies, in extreme cases, where the total replacement of the ligament is required, generates materials that imitate living tissues, studying their physical and mechanical properties and obtaining biomaterials that not only imitate tissue but also behave as biointelligent materials.

The higher the degree of biofidelity, the greater the number of materials that shape, and the greater the geometry of the biomodel, a greater computational resource or more robust specifications (large capacity in RAM memory, graphic cards, faster processors) will be required. This does not mean that it is not possible to generate it in conventional computer equipment. In addition to this, technological advances, and greater access to them, to acquire computer equipment that have better graphics cards and a greater capacity of RAM memory, have allowed to reduce the cost compared to the costs a few years ago.

## Figures and Tables

**Table 1:** Biomodel contacts [36].

	Contact body	Target body
Contact 1: between the cortical bone and trabecular bone of the femur	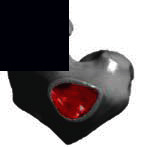	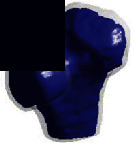
Contact 2: between the cortical bone of the femur and the PCL	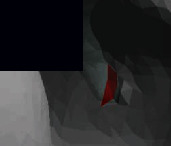	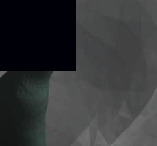
Contact 3: between the cortical bone of the femur and the *ACL*	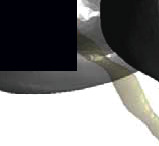	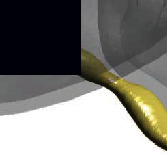
Contact 4: between the cortical bone of the femur and the ELL	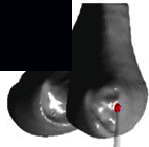	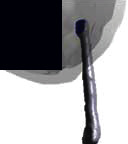
Contact 5: between the cortical bone of the femur and the ILL	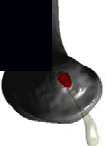	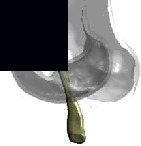
Contact 6: between the cortical bone of the femur and the outer meniscus	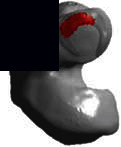	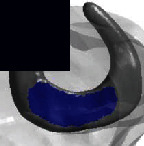
Contact 7: between the cortical bone of the femur and the inner meniscus	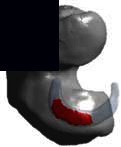	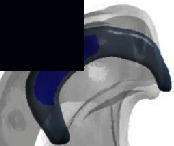
Contact 8: between the cortical bone and the trabecular bone of the tibia	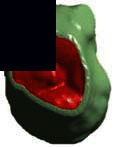	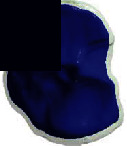
Contact 9: between the cortical bone of the tibia and the PCL	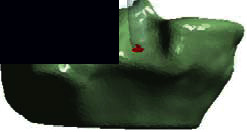	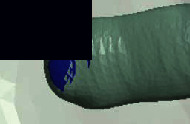
Contact 10: between the cortical bone of the tibia and the *ACL*	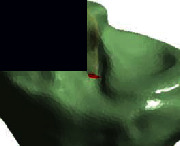	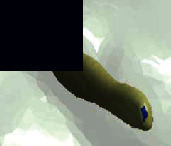
Contact 11: between the cortical bone of the tibia and the ELL	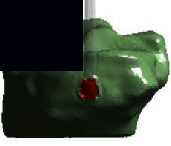	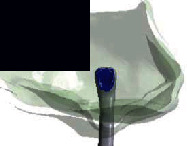
Contact 12: between the cortical bone of the tibia and the ILL	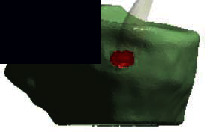	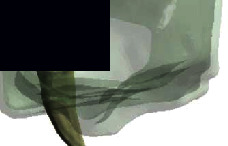
Contact 13: between the cortical bone of the tibia and the outer meniscus	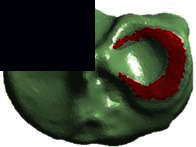	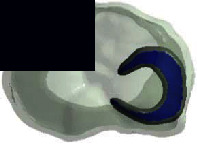
Contact 14: between the cortical bone of the tibia and the inner meniscus	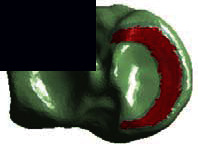	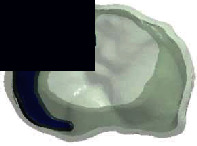

**Table 2:** Mechanical properties used in the analysis [36–42].

Tissues	*Young's* modulus (MPa)	*Poisson's* ratio
Cortical bone	15 000	0.32
Trabecular bone	100	0.3
*ACL*	64	0.45
*PCL*	67	0.45
Lateral ligaments	61	0.45
Meniscus	55	0.3

**Table 3:** Some characteristics of the generated biomodel.

	Control case	Sprain 1°	Sprain 2°	Sprain 3°
Mesh	Tetrahedral solid elements	Tetrahedral solid elements	Tetrahedral solid elements	Tetrahedral solid elements
Meshing	Semicontrolled	Semicontrolled	Semicontrolled	Semicontrolled
Mesh quality	High-order quadratic elements	High-order quadratic elements	High-order quadratic elements	High-order quadratic elements
Nodes	1052629	1054252	1054464	1056490
Elements	612084	612971	613082	614128

**Table 4:** Comparison of compression and tensile stress (MPa) between case studies.

Normal stress	Case 1 Control case		Case 2 *ACL* sprain 1°		Case 3 *ACL* sprain 2°		Case 4 *ACL* sprain 3°	
	Compression stresses	Tensile stresses	Compression stresses	Tensile stresses	Compression stresses	Tensile stresses	Compression stresses	Tensile stresses
*X*-axis	-0.1711	0.2342	-0.1753	0.2437	-0.1568	0.2319	-0.3134	0.1076
*Y*-axis	-0.2051	0.1793	-0.2527	0.1713	-0.2303	0.1615	-0.3447	0.1892
*Z*-axis	-0.3970	0.3572	-0.3852	0.3685	-0.3363	0.3453	-0.5709	0.1196

**Table 5:** Comparison of *von Mises* stresses (MPa) between case studies.

Case 1 Control case		Case 2 *ACL* sprain 1°		Case 3 *ACL* sprain 2°		Case 4 *ACL* sprain 3°	
Min.	Max.	Min.	Max.	Min.	Max.	Min.	Max.
0.0013	0.3811	7 × 10^−4^	0.3728	13 × 10^−5^	0.3399	2.58 × 10^−6^	0.6205

## Data Availability

The data used to support the findings of this study are included within the article.
